# A sensitive method for rapid detection of alkyl halides and dehalogenase activity using a multistep enzyme assay

**DOI:** 10.1186/2191-0855-2-51

**Published:** 2012-09-24

**Authors:** Sebastian Fabritz, Franziska Maaß, Olga Avrutina, Tim Heiseler, Björn Steinmann, Harald Kolmar

**Affiliations:** 1 Institute of Organic Chemistry and Biochemistry, Technische Universität Darmstadt, Petersenstrasse 22, D-64287, Darmstadt, Germany

**Keywords:** Alcohol oxidase, Haloalkane dehalogenase, Haloalkanes, Horseradish peroxidase, Multistage enzyme reaction

## Abstract

A method for the detection of haloalkane conversion to the corresponding alcohols by haloalkane dehalogenases is described. It is based on a multistage enzyme reaction which allows for the analysis of alkyl halides in buffered systems. Irreversible hydrolytic dehalogenation catalyzed by haloalkane dehalogenase DhaA from *Rhodococcus erythropolis* transfers an alkyl halide into a corresponding alcohol that is further oxidized by alcohol oxidase AOX from *Pichia pastoris* yielding a respective aldehyde and hydrogen peroxide easily detectable via the horseradish peroxidase catalyzed oxidation of chromogenic molecules. Due to its high sensitivity (0.025 mM, 0.43 ppm for 1,3-dibromopropane), low expenditure and the ability of handling a large number of samples in parallel, this method is an attractive alternative to existing procedures for the monitoring of both haloalkanes and dehalogenases.

## Introduction

Haloalkanes are toxic (Akers et al., [1999], [Koch and Strobel 1981], Weber et al., [2003]) and mutagenic (Brem et al., [1974]) environmental contaminants ([Koch and Tunger 1981], Yen et al., [2002]). Many bacterial species that are able to degrade such compounds have been described to date. Some of them can even utilize haloalkanes as a sole carbon source (Manickam et al., [2008], Mattes et al., [2008], Torz et al., [2007]). Bioremediation based on the capability of certain microorganisms to dispose halogenated pollutants is a promising and cost-effective technology ([Beeman and Bleckmann 2002], Marzorati et al., [2006], Megharaj et al., [2011], Vlieg et al., [2000]). Therefore, convenient methods are required to determine the activity of dehalogenases in enrichment cultures and to detect alkyl halides in environmental samples.

Methods for the determination of halogenated content in various samples have been established since the early 1950s (Iwasaki et al., [1952]). A common method for the analysis of haloalkanes is gas chromatography combined with flame-ionization/electron capture or mass spectrometric detection ([Arbon and Grimsrud 1990], Curragh et al., [1994], Phillips et al., [2001], van Wijk et al., [2011]). These methods, although providing detailed information about the nature and composition of haloalkanes present in a sample, are technically demanding. More recently, procedures for haloalkane detection and degradation have been reported which rely on enzyme-catalyzed dehalogenation yielding free protons and halides (van [Pee and Unversucht 2003]). Therefore, the majority of current haloalkane assays is focused on the monitoring of proton or halide release. Several pH dependent detection systems rely on chromatic (Holloway et al., [1998], Phillips et al., [2001]) or fluorescent indicators (Bidmanova et al., [2010]) that require weakly buffered or unbuffered aqueous systems. Alternatively, methods enabling direct estimation of halide concentration have been developed as e.g. the classic colorimetric mercury-iron-thiocyanate method (Cirello-[Egamino and Brindle 1995], Iwasaki et al., [1952], Zall et al., [1956]), iodide detection via starch incorporation (Kurtovic et al., [2007]) or quenching of fluorophores by halides ([Marchesi 2003]).

**Figure 1:**
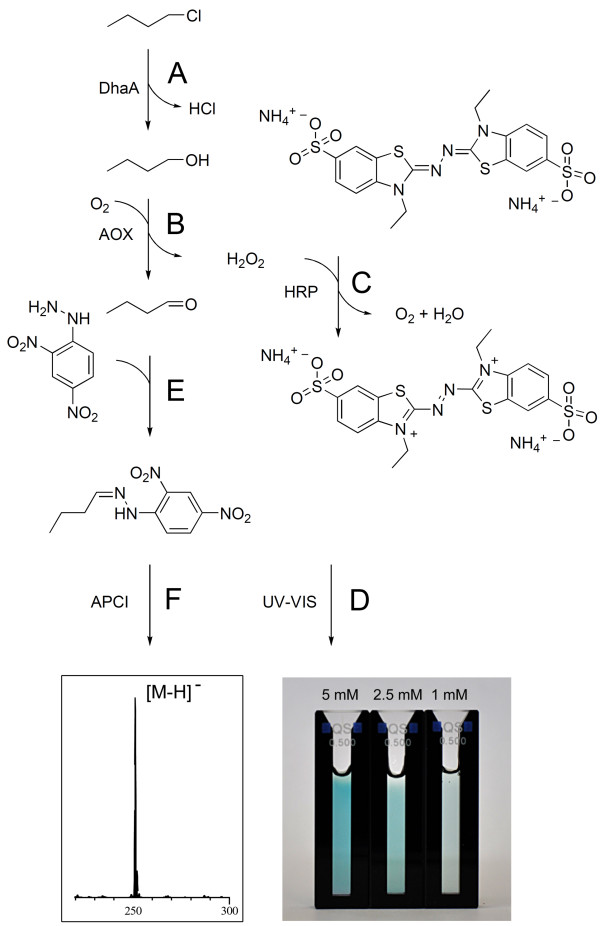
**General scheme of an enzymatic multistep assay for the detection of haloalkanes.** The alcohol formed via dehalogenation (**A**) undergoes an enzymatic oxidation and H_2_O_2_ is liberated (**B**). Afterwards a peroxide-HRP complex oxidizes the chromogen ABTS (**C**) resulting in a measureable increase of absorbance at 405 nm (**D**). The enzymatic formation of primary aldehydes can alternatively be proven by precipitation with 2,4-DNPH (**E**) and a subsequent APCI-MS measurement of the resulting hydrazones (**F**).

Herein, we report an alternative biochemical approach to haloalkane detection based on a set of coupled enzyme reactions. At the first step, a haloalkane is converted into a corresponding aliphatic alcohol by a hydrolytic dehalogenation that is catalyzed by a microbial haloalkane dehalogenase (DhaA) (Figure 1A) (Curragh et al., [1994], Koudelakova et al., [2011], Kulakova et al., [1997], Stsiapanava et al., [2008]). Then, an alcohol is oxidized by an alcohol oxidase (AOX) ([Couderc and Baratti 1980], Sahm et al., [1982], Van der Klei et al., [1990]) to an aldehyde yielding H_2_O_2_ as a detectable by-product (Figure 1B) (Ukeda et al., [1999]). Finally, hydrogen peroxide is used by horseradish peroxidase (HRP) as a redox substrate for the oxidation of different chromogens ([Braithwaite 1976], Delincée and [Radola 1975], [Welinder 1979]), where 2,2’-azino-di-(3-ethyl-benzthiazoline-6-sulphonic acid) (ABTS) is known to be the most sensitive one (Figure 1C and 1D) ([Childs and Bardsley 1975], Porstmann et al., [1981]). In addition, the aliphatic aldehyde formed during AOX oxidation of an alcohol readily reacts with 2,4-dinitrophenylhydrazine (2,4-DNPH, Figure 1E) and therefore allows for both photometric and mass-spectrometric detection of the resulting hydrazone (Figure 1F).

## Materials and methods

### Chemicals and enzymes

The chemicals and enzymes were of analytical grade and used without further purification. Alcohol oxidase (AOX) from *Pichia pastoris* (solution in phosphate-buffered 30% sucrose, 10-40 U/mg) and peroxidase (HRP) from horseradish type VI-A (950-2000 U/mg) were obtained from Sigma-Aldrich (USA). 2,2^′^-Azino-bis(3-ethylbenzothiazoline-6-sulfonic acid) diammonium salt (ABTS) was purchased from Fluka. Ampicillin sodium salt, isopropyl-β-D-thiogalactopyranoside (IPTG), potassium dihydrogen phosphate and dipotassium hydrogen phosphate were obtained from Carl Roth (Germany).

### Expression and purification of haloalkane dehalogenase

The haloalkane dehalogenase gene (*dhaA*) from *Rhodococcus erythropolis* DSM 16550 ([Gray and Thornton 1928]) has been deposited in the GenBank database under accession no. AF060871.1. The gene was isolated by polymerase chain reaction (PCR) and cloned into the pET21d expression vector (Novagen) to yield pET21d-DhaA. The expression vector was transferred into *E. coli* BL21(DE3). Transformed *E. coli* cells were cultured in 3 liters of dYT medium (1% yeast extract, 1.6% Bacto tryptone, 0.5% NaCl) supplemented with 100 μg/mL ampicillin at 37°C and 180 rpm. Expression was induced with 1 mM IPTG when bacterial growth reached an A_600_ of 0.5 and performed for 18 h at 30°C. The cells were harvested by centrifugation, resuspended in potassium phosphate buffer (0.1 M, pH 7.5) and the cell suspension was stored at -80°C for 1 h. Cells were thawed and disrupted using a high-pressure cell disruption system from Constant Systems Limited (United Kingdom). The suspension was centrifuged at 19650 × g for 30 min at 4°C. The enzyme was purified from the supernatant by immobilized metal ion chromatography using Ni-loaded IMAC Sepharose 6 Fast Flow (GE Healthcare) and a step gradient of imidazole as eluent. Purified enzyme was dialyzed against potassium phosphate buffer (0.1 M, pH 7.5) and frozen in aliquots at -80°C until needed.

### Detection of haloalkanes

A quartz cuvette with a path length of 5 mm (type: 104B-QS; Hellma Analytics, Germany) was filled with 500 μL potassium phosphate buffer (0.1 M, pH 7.5) containing the corresponding halogenated compound in desired concentration. 5 μL of ABTS solution in *aqua bidest* (10 mM), 1 μL AOX dissolved in phosphate buffer containing 30% sucrose (1500 U/mL) and 1 μL of a HRP solution in *aqua bidest* (15 kU/mL) was added successively. After an equilibration time of 15 min, 20 μL of purified DhaA in potassium phosphate buffer (~0.32 U/mL) were added. The absorbance was measured at 405 nm using a Shimadzu UV–vis spectrophotometer UV-1650PC over 5 min at ambient temperature.

**Figure 2:**
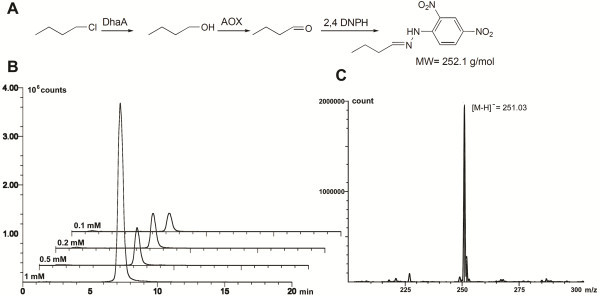
**Detection of haloalkanes via formation of LC-MS (APCI) detectable hydrazones.** General reaction pathway for the hydrazone formation using 2,4-DNPH (**A**), isolated ion currents of the formed hydrazones. The initial 1-chlorobutane concentrations are given (**B**), APCI-MS spectrum of the measured hydrazone (**C**).

The enzymatic conversion of halogenated compounds into corresponding aldehydes was additionally verified using atmospheric pressure chemical ionisation mass spectrometry (APCI-MS, Figure 2). To that end, the reaction mixture of the enzymatic assay was allowed to stand for 2 h and was subsequently acidified with 75 μL concentrated hydrochloric acid to precipitate the enzymes. After 20 min, the suspension was centrifuged and 75 μL of a saturated solution of 2,4-DNPH in concentrated hydrochloric acid was added to 400 μL of the supernatant. After a reaction time of 30 min, 100 μL acetonitrile were added to assure the solubility of the formed hydrazones. 40 μL of the resulting solution were analyzed using a Shimadzu Mass Spectrometer LC-MS 2020 (gradient: 20 to 80% acetonitrile with 0.1% formic acid over 8 min).

## Results

**Figure 3:**
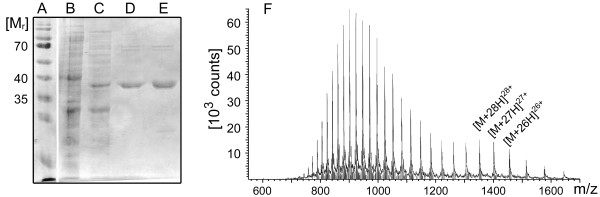
**(A-E) SDS Page of DhaA purification via IMAC.** (**A**) Prestained Protein Ladder, (**B**) potassium phosphate buffer wash, (**C**) IMAC column elution with 20 mM imidazole, (**D**) IMAC column elution with 40 mM imidazole, (**E**) IMAC column elution with 500 mM imidazole; (**F**) ESI-MS of purified DhaA, measured m/z: 1351.17 [M + 28 H]28+, 1401.07 [M + 27 H]27+, 1454.98 [M + 26 H]26+, calculated mass using ApE - a plasmid editor: 37668 g mol^-1^, measured deconvoluted mass 37805.59 g mol^-1^.

For the detection of haloalkanes or haloalkane dehalogenase, respectively, activity via a multistep bioassay, the haloalkane dehalogenase DhaA from *R. erythropolis* was used as a model enzyme. The His-tagged protein was produced via expression in *E. coli*, purified by immobilised metal ion affinity chromatography (IMAC) and analyzed by SDS-polyacrylamide gel electrophoresis (SDS-PAGE, Figure 3, A-E) and electrospray ionization mass spectrometry (ESI-MS, Figure 3G).

**Figure 4:**
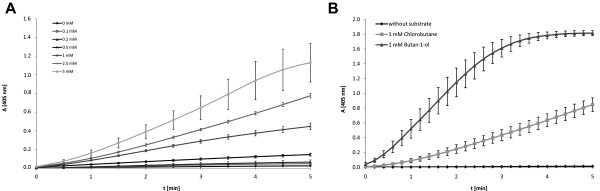
**(A) Kinetic measurement of ABTS oxidation product formation at 405 nm.** Experiments were performed in triplicate. (**B**). Comparison of reaction rates of DhaA/AOX/HRP mediated formation of ABTS using 1-chlorobutane or butan-1-ol as substrate, respectively. Experiments were performed in triplicate with 1 mM 1-chlorobutane and butan-1-ol.

Addition of DhaA together with commercially available and inexpensive AOX and HRP to haloalkanes allows for the subsequent oxidation of the alcohol (generated by DhaA-catalyzed halide abstraction) into a corresponding carbonyl derivative and utilization of the resultant hydrogen peroxide as a cosubstrate of HRP with subsequent formation of an analytical signal by ABTS oxidation. Formation of aldehydes by coupled reaction of the dehalogenase and the alcohol oxidase was confirmed via APCI-MS monitored conversion of the aldehydes to hydrazones using 2,4-dinotrophenylhydrazine (Figure 2). Likewise, the increase of H_2_O_2_ liberation was detected by HRP and ABTS as a substrate (Figure 4A). In principle, DhaA activity and halide content can be measured via endpoint determination or by the rate of chromophore formation. For quantification of dehalogenase activity it is essential that the DhaA-controlled dehalogenation towards a corresponding alcohol is a rate-determining step. Therefore, a set of parallel experiments to compare reaction rates of DhaA- and AOX-catalyzed transformations was performed with 1 mM 1-chlorobutane and butan-1-ol, respectively. Kinetic assays confirmed that the AOX-driven reaction has proceeded enormously fast compared to the DhaA-mediated dehalogenation using 1.5 U AOX and 6.4 mU DhaA (Figure 4B). Since HRP was used in significant excess, its influence on the overall velocity was negligible (data not shown).

To develop appropriate experimental conditions under which the initial reaction velocity of the coupled three-step enzyme reaction directly corresponds to the initial substrate concentration, 1-chlorobutane was used as a model substrate at different concentrations. To obtain reliable values, measurements were performed in triplicate and the initial reaction rates were determined from the reaction progress curve using a time-frame ranging from 2 to 4 minutes. After substrate addition the samples were preincubated (15 min) as an initial delay in reaction progress has been observed.

**Figure 5:**
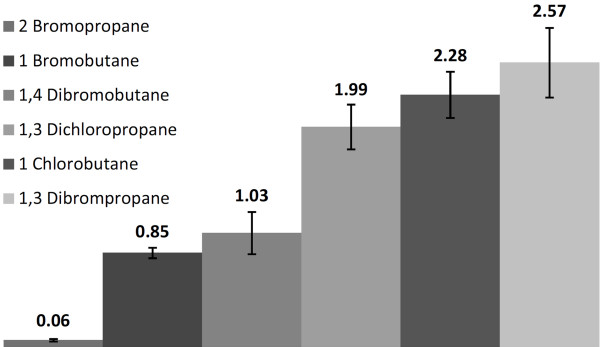
**Comparison of reaction velocities of ABTS oxidation product formation as a result of dehalogenation using various haloalkane substrates at 1 mM concentration.** The numbers are the initial reaction rate in nmol/min with error bars from three measurements.

To evaluate the feasibility of this method, a set of haloalkane substrates for *R. erythropolis* DhaA was chosen (Koudelakova et al., [2011]). They varied in the length of the alkyl chain (C_3_ and C_4_) as well as the character (Cl, Br), position (primary, secondary), and amount (mono-, disubstituted) of halogen atoms. As expected, the enzyme displayed the highest activity with short-chain mono- and dibromo derivatives while the secondary haloalkane compound was only slowly converted into the corresponding alcohol (Figure 5).

**Figure 6:**
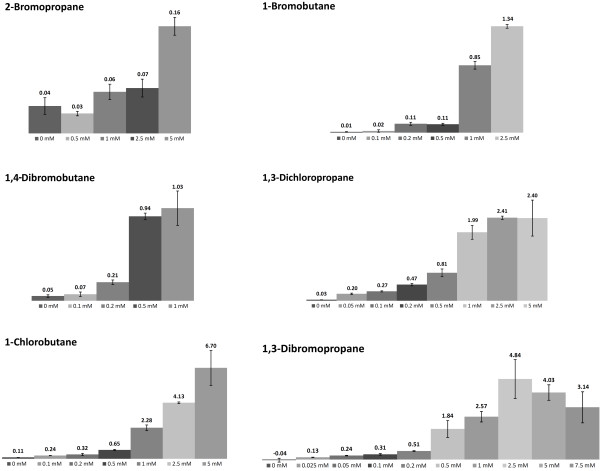
**Concentration dependent initial velocities of ABTS oxidation product formation of different substrates.** Numbers indicate the reaction rate of dehalogantion in nmol/min with error bars from three measurements.

Although 2-bromopropane could only be detected at 5 mM concentration (85.4 ppm), the assay was sensitive enough to analyze the content of primary monosubstituted and disubstituted haloalkanes. The fastest turnover and highest detection sensitivity was found for 1,3-dibromopropane that was measured in micromolar concentrations (0.025 mM, 0.43 ppm, Figure 5 and 6).

## Discussion

Haloalkane dehalogenases have been isolated from a number of species and also from uncultivated environmental samples using polymerase chain reaction ([Kotik and Famerova 2012]). Each specific dehalogenase can be expected to have its own characteristic substrate specificity, enantioselectivity and product inhibition properties. For detection of various haloalkanes we have chosen the *R. erythropolis* DhA since it is well characterized and it has been reported to display broadened substrate specificity (Koudelakova et al., [2011], Pavlova et al., [2009]). Moreover, this parameter can be further extended even towards acceptance of mono-, di-, and trichloro-substituted substrates by enzyme engineering (Banas et al., [2006], Pavlova et al., [2009]).

The coupled triple enzyme reaction described here allows fast simple and sensitive detection of haloalkanes but depending on the nature of the sample to be analysed several potentially limiting conditions have to be carefully considered. It has been reported that certain dehalogenases are inhibited by halides (Schindler et al., [1999]). In the case when *R. erythropolis* DhaA is used, presence of halide salts in concentrations up to 80 mM should not disturb the reaction (Schindler et al., [1999]). It should also be noted that the signal generation in our method can similarly be triggered by traces of alcohols in the sample. In this case, it may be advisable to preincubate the analyte solution with AOX and a catalase to oxidize the alcohol to the corresponding aldehyde and to remove the hydrogen peroxide generated. Subsequent inactivation of catalase, e.g. by addition of 3-amino-1,2,4-triazole or 4-hydroxypyrazole (Mac[Donald and Pispa 1980], Margoliash et al., [1960]), allows one to apply the standard procedure described above. Like other methods relying on the determination of halide content (Holloway et al., [1998], Kurtovic et al., [2007], [Marchesi 2003]), this assay does not allow to distinguish between individual alkyl halides if a multicomponent mixture of haloalkanes is present in a sample. Since different halogenated alkanes give rise to distinct sensitivity and reaction rates, knowledge of the haloalkane composition of a sample would be required. It can be obtained by using e.g. GC-MS analytics via the generation of an equivalent reference sample for calibration purposes.

It should also be noted that with this triple enzyme assay no linear correlation exists between analyte content and initial velocity of ABTS formation over the range of 0 to 5 mM substrate concentration (Figure 6). Several reasons may account for this finding. At low haloalkane concentration, the accuracy of the measurement may be limited due to the fact that a fraction of the primary aldehydes could react with primary amines of the enzymes present in the reaction mixture via Schiff base formation ([Shan and Hammock 2001]) which would impede oxidation by HRP.

Obviously, besides measurement of haloalkane content in a sample, the coupled assay can also be used for the determination of haloalkane dehalogenase activity, e.g. in an enrichment culture, using halogenated hydrocarbon substrates for which the enzyme of interest displays the highest catalytic efficiency. The detection system we report here is nontoxic, works in a buffered system, is rapid and, due to the enzyme-mediated chromophore formation, highly sensitive. The assay does not require sophisticated machinery (chlorimeter, special electrodes, etc.), and the enzymes apart from haloalkane dehalogenases are inexpensive and commercially available. Furthermore, the established multistage enzyme reaction can be considered as a modular system for haloalkane detection. The usage of different haloalkane dehalogenases ([Janssen 2004], Koudelakova et al., [2011]) or DhaA variants that have been optimized by directed evolution (Pavlova et al., [2009]) should result in an extension of accepted substrates if required.

Recently, an enzyme-based method for the detection of halogenated hydrocarbons that relies on an enzymatic fibre-optic biosensor has been reported with similar detection limits (Bidmanova et al., [2010]). The assembly of such a device needs special equipment and fine-tuned immobilisation chemistry. Nevertheless, it has the inherent capability of continuous *in situ* measurement. Another interesting approach was developed by Marchesi ([Marchesi 2003]). The assay is based on the fluorescence quenching of 6-methoxy-*N*-(3-sulfopropyl)-quinolinium by halides. This elegant methodology that allows one to detect halide concentrations in the range of 1-500 mM is restricted to the samples where halide salts are absent since they quench the fluorophore. The approach described here is at least as sensitive as other methods and, depending on the nature of the haloalkane substrate and enzyme, may allow for an even lower detection limit.

In conclusion, we have developed a fast, simple and sensitive detection of haloalkanes and haloalkane dehalogenase activity based on coupled enzymatic reactions. This method may be useful for the detection of halogenated pollutants in environmental samples or for the detection of haloalkane dehalogenase activity e.g. in enrichment cultures or to control dehalogenase activity during bioremediation. Using DhaA from *R. erythropolis* as a model enzyme, we showed that the rate-determing step of the multistep assay was dehalogenation of a haloalkanes substrate. Detection can be conducted either “on-bench”, with green colour of a sample indicating the enzymatic conversion of haloalkanes, or, more precisely, by photometric monitoring of the formation of an ABTS oxidation product. Our method allows for the detection of enzyme-mediated haloalkane conversion in buffered systems and, depending on the dehalogenase used, in samples that may contain inorganic halides. High sensitivity (0.025 mM, 0.43 ppm for 1,3-dibromopropane), low expedition, and possibilities to vary haloalkane dehalogenase towards broadened substrate tolerance makes this method a versatile alternative to existing procedures.

## Competing interests

The authors declare that they have no competing interests.
